# Supplementary material for: Varietal and seasonal differences in the effects of commercial bumblebees on fruit quality in strawberry crops
Source: Agric Ecosyst Environ. 2019 Sep 1;281:124–33. doi: 10.1016/j.agee.2019.04.007 (PMC6686987; doi:10.1016/j.agee.2019.04.007)
Supplement: Supplementary file 1 [file mmc1.docx]

**Varietal and seasonal differences in the effects of commercial bumblebees on fruit quality in strawberry crops**

Callum D. Martin^a,*^, Michelle T. Fountain^b^ & Mark J. F. Brown^a^

*^a^School of Biological Sciences, Royal Holloway University of London, Egham, Surrey, United Kingdom, TW20 0EX*

*^b^NIAB EMR, East Malling Research, East Malling, Kent, United Kingdom, ME19 6BJ*

*****Corresponding author: C. D. Martin, School of Biological Sciences, Royal Holloway University of London, Egham, Surrey, United Kingdom, TW20 0EX.

*E-mail address*: [Callum.Martin.2014@live.rhul.ac.uk](mailto:Callum.Martin.2014@live.rhul.ac.uk) (C. D. Martin)

*Telephone*: +44 (0) 7778683632 (C. D. Martin)

**Abstract**

Both wild and managed pollinators significantly contribute to global food production by providing pollination services to crops. Colonies of commercially-reared honey bees and bumblebees are two of the largest groups of managed pollinators. Bumblebees in particular are increasingly used on soft fruit crops, such as strawberry, an economically important crop globally. Despite the use of commercial bumblebees in strawberry crops, there is little quantitative evidence that they provide a benefit to farmers. Given the negative impacts that commercial colonies can have on wild bee populations, it is vital that the benefits of commercial bumblebees are quantified, so reasoned management decisions can be made that provide maximum benefit to both farmers and wild bees. In this study, commercial colonies of the UK native subspecies *Bombus terrestris audax* were placed into June-bearer (flowering March-April, varieties ‘Malling Centenary’ and ‘Flair’) and everbearer (flowering May-June) strawberry polytunnels on a soft-fruit farm in the south east of England, and opened and closed at weekly intervals. The flower-visiting assemblage inside polytunnels was quantified, and fruit was harvested and quality assessed. In the June-bearer variety Malling Centenary, the presence of commercial bumblebees increased the amount of high commercial grade fruit by 25%. In contrast, no benefit of commercial bees on pollination or fruit quality was observed in the June-bearer variety Flair and the everbearer crop. The increase in quality of fruit in the Malling Centenary crop may be driven by the higher *B. terrestris audax* flower visitation rates seen in this crop in combination with varietal differences in pollination dependency. The number of flower visits by wild pollinators was not a well-supported predictor of strawberry quality, thus the benefit they provide in this system remains to be elucidated. The results presented here suggest that commercial bumblebees can greatly increase the quality and subsequent value of a strawberry crop, when deployed on a suitable variety at a time when wild pollinator numbers are low. However, the results also raise the possibility that commercial colonies do not always provide the benefits to strawberry crops that they are thought to. For growers to make informed decisions on commercial bumblebee use further research is required into the effect of commercial bumblebees on the major strawberry varieties, in different locations and seasons. This study is an important step in gaining this understanding.

**Keywords:** pollination, *Fragaria*, Nitidulidae, commercial *Bombus terrestris audax*, fruit quality, ecosystem service

**1. Introduction**

Entomophilous crop pollination is a valuable ecosystem service that contributes to human health, wellbeing, and global food security (Aizen et al., 2009; Klein et al., 2007). 75% of the 115 major global crop species depend to some degree on insect pollination that is provided by both wild and managed pollinators, and many of the most insect-dependent crops provide humans with valuable sources of micronutrients (Eilers et al., 2011; Klein et al., 2007; Wang and Ding, 2012). Bees are one of the most important pollinator groups (Calderone, 2012; Klein et al., 2007), partially due to their large scale management in order to support crop pollination. Globally, honeybees are the dominant managed pollinator (Calderone, 2012; Klein et al., 2007), but managed bumblebees are of increasing importance, being superior pollinators in some crop types and possessing the ability to forage in cooler, windier weather (Berger et al., 1988; Goodell and Thomson, 2007; Stanghellini et al., 1998, 1997; Thomson and Goodell, 2002).

Bumblebees were first commercially produced in the mid 1980’s, and colonies were used primarily in greenhouse tomato crops (Velthuis and van Doorn, 2006). Prior to the introduction of commercial bumblebees, tomatoes had to be mechanically pollinated, so the use of bees drove down labour costs and also improved yield and quality of the fruit (van Ravestijn and Nederpel 1988; Velthuis and Van Doorn 2006). This success, combined with reports of pollinator declines and subsequent fears of pollen limitation in crops, has led to growth in the trade of commercial bumblebees (Potts et al. 2010, Lye et al. 2011). Colonies are now mass produced in several rearing facilities, and in 2006 it was reported that over 1 million colonies were shipped worldwide (Velthuis and van Doorn 2006). They are increasingly being used in crops other than tomatoes, some of which are grown in polytunnels and open fields (Velthuis and van Doorn 2006). In the UK, for example, around 15,000 colonies per year are used on soft fruit farms (Goulson 2009).

Despite the beneficial pollination services commercial bumblebees can provide, there are negative impacts associated with the trade in commercial bumblebees. Among these are competition with local species (Ings et al., 2006; Inoue et al., 2008) and disease spread (Colla et al., 2006; Goka et al., 2006; Graystock et al., 2013; Schmid-Hempel et al., 2014). Indeed, strong evidence of pathogen spill-over from commercial to wild bumblebee populations has been observed in both Europe and The Americas (Colla et al., 2006; Murray et al., 2013), and in South America is thought to be a leading cause in the severe decline of a native bumblebee species (Schmid-Hempel et al., 2014).

Given the negative impacts associated with the commercial bumblebee trade, it is important that bumblebees are used responsibly, and only on crops for which there is supporting evidence of beneficial pollination services. Experiments investigating bumblebee pollination are often done in greenhouse crops, where wild pollinators have very limited or no access to the crop, and where wind pollination is minimal (Dogterom et al., 1998; Shipp et al., 1994; Zhang et al., 2015). Other studies have been done on a small scale, investigating pollen deposition during one or more flower visits, and it is unclear how the results extrapolate to the much larger scale of a farm (Javorek et al., 2002; Thomson and Goodell, 2002). Some field trials have been done at a larger scale in polytunnel or open field crops, and have shown the addition of commercial bumblebees to increase yield and fruit set in blueberry (Desjardins and De Oliveira, 2006; Stubbs and Drummond, 2001), and increase yield in raspberry (Lye et al., 2011). A study on apple orchards in Israel suggested that not only does the addition of bumblebees directly increase the pollination of the crop, but that it can also alter the foraging behaviour of other pollinators on the crop, which then causes further alterations to pollination services (Sapir et al., 2017). However, in pumpkin (Petersen et al., 2013), cranberry (Hicks and Sircom, 2016) and strawberry (Trillo et al., 2018), the addition of commercial bumblebees has been shown to have no beneficial effect on crop yield or quality. This casts doubt upon the use of commercial bumblebees in these crop systems.

Strawberry (*Fragaria x ananassa* DUCH) is a major soft fruit crop, worth £284 million in the UK in 2015 (DEFRA, 2015). Several bee species, both managed and wild, have been shown to be effective pollinators of strawberry (Connelly et al., 2015; Dimou et al., 2008; Klatt et al., 2014; MacInnis and Forrest, 2019). The proportion of the strawberry pollinator community that is made up of bumblebees varies. In some cases they only make up a small proportion of the community (Ahrenfeldt et al., 2015; Klatt et al., 2014), but in others, they are the dominant pollinator group (Wietzke et al., 2018), including in cases when commercial bumblebees are deployed (Feltham et al., 2015). The use of commercial bumblebees on strawberry farms in the UK is widespread, but the contribution of both commercial bumblebees and wild pollinators to strawberry pollination has not been investigated. Recent evidence from southern Spain suggests that commercial bumblebees do not provide any benefit to strawberry crops (Trillo et al., 2018). Thus, there is an urgent need to test their effectiveness in the UK and in other strawberry growing regions, where different environmental conditions and farming practices may cause results to vary compared to those observed in Spain.

This study examined the contribution to crop pollination and fruit quality made by commercial bumblebees on a strawberry farm in the south east of England. Commercial colonies were placed into June-bearer (varieties ‘Malling Centenary’ and ‘Flair’) and everbearer crops, and were or were not allowed to forage on the crop during specific time periods. Fruits that were pollinated during these time periods were picked and quality assessed to examine the impact commercial bees were having on fruit quality. The wild pollinator community was also surveyed to assess its potential for providing pollination services to the crop.

**2. Methodology**

**2.1. Study species**

**2.1.1. *Fragaria x ananassa* DUCH (strawberry)**

Strawberry is a hybrid species that is cultivated around the world for its fruit (FAOSTATS). Strawberry flowers are hermaphroditic and self-fertile, and are thus able to set fruit without animal mediated pollination. However, bee pollination increases the likelihood of each pistil receiving a pollen grain from the stamens, which results in increased fruit yields and quality (Dimou et al., 2008; Klatt et al., 2014). If the flower is not fully pollinated, i.e., not all pistils receive a pollen grain, it can lead to the development of deformed fruit, which have a reduced market value (European Commission, 2011; Klatt et al., 2014). It is for this reason that commercial bumblebee colonies are regularly used to provide pollination services to strawberry crops.

In a polytunnel environment in the south of England, the lifespan of a strawberry flower is approximately 3-5 days (Whitehouse pers. comm.). After this time the petals begin to senesce and drop and the receptacle begins to form a fruit.

**2.1.2. *Bombus terrestris audax***

The commercial bumblebees placed into the strawberry crop on the farm were *Bombus terrestris audax*. This *B. terrestris* subspecies is native to the British Isles (Rasmont et al., 2008), and is currently the only commercially produced bumblebee species used in the United Kingdom. Commercial colonies are typically placed into a crop when they reach a size of 50-100 individuals, although this can vary depending on the supplier. They are then estimated to be able to provide pollination services for the next 6-8 weeks.

**2.2. Field site**

The fieldwork was carried out at Kelsey Farms, an 80-hectare soft fruit farm in Kent in the South East of England (latitude: 51.288694, longitude: 1.183766). The landscape surrounding the farm is dominated by pasture and arable crops (predominantly cereal crops), with some small villages and patches of mixed deciduous woodland (see supplementary figure S1 and supplementary tables S1 and S2 for land use map and further details of the area surrounding the farm). No managed honey bee hives were used on the farm, but it is possible that some were present in the surrounding landscape during the experiment.

Two experiments were done on the farm, the first ran for 6 weeks from 21^st^ March to 29^th^ April 2016 and was done in two varieties (‘Malling Centenary’ and ‘Flair’) of June-bearing strawberries. The second ran for 8 weeks from 9^th^ May to 1^st^ July 2016 and was done in a single variety of everbearing strawberry. Due to proprietor constraints, the name of the everbearing variety cannot be released, thus, from here on it is referred to as ‘Proprietary variety 1’. June-bearing strawberries flower earlier in the season and have a shorter flowering period than everbearers, which can flower throughout the summer months. The strawberries were grown in irrigated coir grow bags on a table-top system in polytunnels; the predominant growing system in the UK. During the June-bearer experiment, the ends of the tunnels were open, meaning insects could enter and leave the tunnels. Both the ends and sides of the polytunnels were open for the duration of the everbearer experiment.

In the June-bearer experiment, 9 *B. terrestris audax* colonies were obtained from Biobest. The supplier states that these colonies arrive with approximately 60-80 bees. These colonies were spread across 3 fields (3 colonies in each field) on the farm. The 3 fields were 0.94, 1.41, and 1.26 hectares in size, and contained plants at a density of 47,000 plants/ha. The closest colonies in separate fields were separated by approximately 125m. Fruit were sampled from a sampling area around each colony (see supplementary material figure S2 for layout of sampling areas). Each sampling area consisted of the tunnel with the colony inside, and the two tunnels either side which did not contain colonies (see section ‘2.3. Strawberry marking and collection’ for further details on fruit collection). Ideally, all sampling areas would have been the same size, but this was not possible as the polytunnels in the 3 fields varied in size. In the June-bearer experiment, the size of the sampling areas were between 0.16-0.21 ha, with the majority being between 0.16-0.19 ha. Thus, within each sampling area of 3 polytunnels, the colony densities were between 4.76-6.25 colonies/ha, with the majority being between 5.3-6.25 colonies/ha (see supplementary table S3 for colony densities in each sampling area). This is close to the 6 colonies/ha density recommended by the supplier, and similar to the densities used on other strawberry farms. In two of the sampling areas (sampling areas 3 and 6 on supplementary figure S2), only 2 tunnels were sampled, the tunnel containing the colony and one adjacent tunnel. This was done because these tunnels were much longer, and so to keep the colony density within the sampling area as close to 6 colonies/ha as possible, one fewer tunnel was sampled.

In the everbearer experiment 12 colonies were placed in 4 fields (3 colonies in each field). The 4 fields were 3.94, 1.64, 2.44, and 1.90 ha, and contained plants at a density of 53,000 plants/ha. In the everbearer experiment the size of the sampling areas were between 0.16-0.20 ha (see supplementary figure S3 for layout of sampling areas). The closest colonies in separate fields were separated by approximately 95m. 9 of the 12 sampling areas were between 0.160-0.165 ha and had colony density of 6.00-6.25 colonies/ha, however, one field had slightly longer polytunnels meaning that the 3 remaining sampling areas were 0.20 ha with a colony density of 5 colonies/ha (see supplementary table S3 for colony densities in each sampling area). Across June- and everbearers, colonies were placed in the centre of the polytunnels.

The fields were separated by >5m high Alder hedge rows, which act as wind breaks to reduce damage to the crop and polytunnels. In order to have strawberries that were and were not pollinated by commercial bumblebees, the colonies were opened and closed on a weekly cycle for the duration of the experiment. When the colonies were open, both the commercial bees and wild pollinators were able to forage on and pollinate the strawberry crop, but when closed the bees had to remain in the nest, and the crop could only be pollinated by wild pollinators. This meant we had two groups of fruit at the end of the field experiment, one group which could have been pollinated by commercial bumblebees and another group which could not. Within fields, all colonies were kept on the same opening and closing cycle, but between fields colonies were on opposing cycles (i.e. in one field all the colonies would be closed, whilst at the same time in another field they would be open). When colonies transitioned from being open to closed, they were closed at nightfall to reduce the chance of trapping any workers outside the colony. Bees had access to sugar solution from a reservoir beneath the nest at all times, as this is standard practice used by commercial growers. During the weeks when colonies were closed, they were supplemented with approximately 20g of pollen (Biobest UK Ltd) to allow continued growth of the colony.

**2.3. Strawberry marking and collection**

10 recently opened strawberry flowers were marked in the sampling area around each colony every week. Flowers were marked two days into the treatment periods, so they could not have been visited the previous week as they were still closed. Flowers were judged to have recently opened if their anthers contained large amounts of bright yellow/orange pollen, there was no darkening or discolouration of the pollen or the petals, and the receptacle showed no signs of fruit formation (see supplementary figure S4 for example of a recently opened flower). Marking flowers in this state meant they would be receptive during the time that commercial bumblebees were either able or not able to forage on them, but ensured they would not be receptive the following week when the state of the colony was reversed. 5 of the 10 marked flowers in each sampling area were evenly spaced along the same polytunnel as the commercial colony, and a further 5 were in the two tunnels either side of the tunnel containing the colony (3 marked in one tunnel and 2 in another). In the case of the two longer polytunnels in the June-bearer experiment (sampling areas 3 and 6 on supplementary figure S2) 5 flowers were marked in the same tunnel as the commercial colony, and the other 5 were marked in one adjacent polytunnel. The flowers were marked with a small twist of coloured wire, different colours were used to represent different weeks of the experiment, and different weeks of the experiment were associated with times when colonies were open or closed.

Marked berries were picked just before they fully ripened to reduce the chance of farm employees harvesting them. Upon picking, the growth position (primary, secondary, tertiary, quaternary) of each fruit was noted following Darrow (1929). Noting the growth position is important because fruits from later growth positions are usually smaller in size (Tuohimetsä et al., 2014; Webb et al., 1974).

At the end of the experiment, there were a group of berries from times when colonies were open that could have been pollinated by commercial bees and wild pollinators, and a group of berries that could only have been pollinated by wild pollinators. Totals of 382 (207 and 175 from when colonies were open and closed respectively) and 826 (416 and 410 from when colonies were open and closed respectively) tagged fruit were picked from the June-bearer and everbearer experiment respectively. The growth positions of all berries were known. All fruits were stored at -20°C for later quality assessment (see section ‘2.6. Strawberry quality assessment’ for details).

**2.4. Recording flower visitation rate**

Every week 30 minute transects were walked along the centre of each polytunnel that contained a colony, but not along the adjacent tunnels in the same sampling area. All transect walks were conducted between 08:00-19:00 and each week the order of sampling was changed to ensure that individual polytunnels were sampled at different times of day every week. Every individual insect that was observed visiting strawberry flowers within 1 metre either side of the transect line was recorded and identified into one of the categories defined in Table 1. If it was necessary to take a collection of an insect, the transect time was paused whilst the collection was done. Workers of *B. terrestris audax*, *B. lucorum, B. magnus* and *B. cryptarum* were grouped together due to the difficulty of reliably separating them in the field. A flower visit was defined as an individual being present on any part of the flower. The flower visitation rate was reported as visits hour^-1^.

**Table 1.** *Taxonomic groups that all flower visitors were placed into. When possible, individuals were identified to lower levels within each category.*

Dye dispensing boxes were attached to the entrance/exit of every commercial colony to make commercial *B. terrestris* *audax* and wild *B. terrestris audax* visually distinguishable. The design was adapted from Martin et al (2006). The dye dispensers worked by dispensing a small amount of non-toxic coloured powder dye onto the dorsal surface of the thorax of every bee that exited the colony. The coloured dye served to identify bees from commercial colonies during transect walks. The dye dispensing method was only used for the June-bearing experiment for two reasons. Firstly, it proved to be an unreliable method for distinguishing between commercial and wild *B. terrestris audax*, as we believe that the commercial bees readily groomed the powder dye off their bodies. Secondly, deposits of dye were found on the surface of a fruit, meaning we could not use this method in a commercial farm setting. Hence, we do not report results from this part of the experiment.

**2.5. Colony activity, weight and quality measures**

Colony activity surveys were done during periods when the colonies were open (once every 2 weeks). The entrance/exit of the nest was observed for 15 minutes and every instance of a bee entering or exiting was recorded.

Colonies were weighed at the start of the experiment and during the time periods when they were closed (once every 2 weeks) when all the bees were present in the colony.

**2.6. Strawberry quality assessment**

Each fruit was assigned a classification (“extra class”, “class 1”, “class 2”, or “class 3”) based on deformations and areas of tightly clustered achenes (see supplementary material figure S5 for examples of strawberries from each commercial grade classification), and following EU marketing guidelines (European Commission, 2011). Fruits are placed into ‘extra class’ if they are highly symmetrical and possess no deformations or clusters of achenes and are greater than 25mm in diameter. Class 3 fruits are highly asymmetrical and deformed, and have tightly clustered achenes. Classes 1 and 2 fall in between these two extremes, and must have a diameter of at least 18mm. Extra class and class 1 fruits are the highest commercial grades and consequently of the greatest market value, class 2 fruits have a reduced market value, and class 3 fruits are unmarketable. Although extra class and class 1 fruit can be separated as mentioned above, in practice they are often combined (Klatt et al., 2014).

The diameter of each fruit was measured to the nearest hundredth of a millimetre at its widest point with digital calipers (Mitutoyo Digimatic Caliper), and each fruit was weighed to the nearest hundredth of a gram.

Finally, the fruit was placed in a food processor (Tefal Minipro 500W) and blended for 10-15 seconds with 100ml distilled water. Fertilised achenes are heavier than water and so sink to the bottom. In contrast, unfertilised achenes float at the surface (Klatt et al., 2014). This separation allows for a very direct measure of pollination success. A further 100-200ml of distilled water was added to the solution in order to create a greater degree of separation between the unfertilised and fertilised achenes, and any achenes that were stuck to the lid, blades or sides of the food processor were washed back into the mixture. The unfertilised seeds were removed from the surface and counted. The water was then very slowly drained into another container, leaving the sunken fertilised seeds to be counted.

**2.8. Statistical analyses**

**2.8.1. *Bombus terrestris audax* flower visitation**

All statistical analyses were done using ‘R’ programming software (R Core Team, 2018). Generalised linear mixed effects models from the package ‘lme4’ (Bates et al., 2017), were used to analyse the number of *B. terrestris audax* flower visits in the June-bearer crop. Poisson error structures were used as the data were counts. In the everbearer crop, negative binomial generalised linear mixed effects models, from the package ‘glmmADMB’ (Skaug et al., 2016), were used to account for overdispersion. The fixed effects included in the models were ‘colony status’ (whether the colony was open or closed), the strawberry variety, and temperature. Temperature and humidity were both measured to be included in models as control variables, as both are known to affect insect foraging (Taylor, 1963). However, humidity was omitted from our models due to its high degree of collinearity with temperature. The random effects included the identity of the polytunnel crossed with the sampling week, to reflect that each polytunnel was repeatedly sampled each week. Ideally ‘field identity’ would have been included in the random effects structure, but the number of levels of ‘field identity’ was too low (3 June-bearer fields and 4 everbearer fields) (Bolker et al., 2009). Consequently, field identity was moved to the fixed effects structure for the everbearer analysis, but this could not be done for the June-bearer analysis, as field identity was strongly correlated with strawberry variety, owing to varieties being grown in separate fields.

**2.8.2. All pollinator flower visitation**

Negative binomial generalised linear mixed effects models, from the package ‘glmmADMB’ (Skaug et al., 2016), were used to analyse the number of wild pollinator visitation events and to investigate whether commercial *B. terrestris audax* presence or visitation abundance was influencing wild pollinator visitation. Negative binomial models were used to account for overdispersion. The covariables included in the models were ‘number of *B. terrestris audax* flower visits’ and temperature. The random effects structure was the same as in the *B. terrestris audax* flower visitation models (see section 2.8.1).

**2.8.3. Strawberry quality**

Linear mixed effects models, from the package ‘lme4’ (Bates et al., 2017), were used to analyse the proportion of fertilised achenes per fruit, fruit weight and fruit diameter. Cumulative link mixed models, from the package ‘Ordinal’ (Christensen, 2017), were used to analyse the strawberry classification variable, these models are suitable for handling ordinal response variables.

The covariables included in the models were ‘colony status’, the ‘growth position’ of the fruit (primary, secondary, tertiary or quaternary), and the Nitidulidae beetle and wild pollinator abundances recorded from transects. The growth position of the fruit was included, as this is known to have a large effect on strawberry quality and so should be taken into account (Tuohimetsä et al., 2014; Webb et al., 1974). Nitidulidae abundance was initially part of the wild pollinator abundance variable. However, the beetles were so numerous and recent evidence suggests that they can have a negative effect on fruit quality (Castle et al., 2019), thus, we treated them as a separate variable to investigate what, if any, effect they were having on strawberry quality in our study system. For the analysis of the June-bearing strawberries, the strawberry variety was also included as a covariable since two varieties were sampled. An interaction term between colony status and variety was also included to investigate whether the two June-bearer varieties responded differently to the presence of commercial bumblebees. Inclusion of such a term was not necessary when analysing the everbearing strawberries, as only one variety was sampled. Field identity was included as a fixed effect in the analysis of the everbearer strawberries for the same reason as stated in section 2.8.1.

The random effects structures of all the strawberry quality models were the same. They all included the identity of the colony crossed with the sampling week.

For all response variables, candidate models were compared using an information theoretic approach. Candidate models included all possible combinations of covariables. A ‘null model’, which only included the intercept as a predictor, was also included in model comparison. The Akaike Information Criterion corrected for small sample sizes (AICc) was used to compare models, those with the lowest AICc were judged to be the best fitting (Johnson and Omland, 2004). If several models were within two AICc units of the optimal model (model with lowest AICc), then parameter estimates were obtained by model averaging the best set of models (Δ2AICc set) using the ‘MuMIn’ package (Bartoń, 2017; Johnson and Omland, 2004). Models were validated by visual inspection of plots of the residuals plotted against the fitted values. Overdispersion and underdispersion were assessed by examining the ratio of the residual deviance to the residual degrees of freedom. Overdispersion was also tested using the R function ‘overdisp_fun()’. Models were not over-dispersed and there was no collinearity between variables used.

**2.8.4. Colony weight and activity**

The weights and foraging activity levels of colonies from the June- and everbearing experiments were aggregated for each experiment and compared using Wilcoxon-Mann-Whitney tests.

**3. Results**

In the June-bearer experiment, 27 hours of transect walks were completed during which 574 strawberry flower visits were observed. A total of 382 tagged fruit were recovered for quality assessment, 207 from when colonies were open and 175 from when colonies were closed. For the everbearer experiment, 48 hours of transect walks were completed during which 5176 flowers visits were observed. 826 tagged fruits were picked, 416 from when colonies were open and 410 from when colonies were closed.

**3.1. *Bombus terrestris*** ***audax* flower visitation**

In the June-bearing strawberry crop, colony status was a strong predictor of *B. terrestris audax* visitation rate to strawberry flowers, featuring in both of the Δ2AICc models (Table 2). When colonies were open, *B. terrestris audax* visitation rate to strawberry flowers was higher than when colonies were closed (Figure 1; estimate = 1.43; 95% confidence intervals = 0.96 – 1.90). Strawberry variety was not a strongly supported predictor of *B. terrestris audax* visitation, suggesting that bumblebees did not strongly prefer one variety over the other. Models including temperature were not well supported, indicating that this variable was not an important predictor of strawberry flower visitation. However, these trends were not evident in the everbearing crop (Figure 2). Here, colony status was not a good predictor of flower visitation (ΔAICc to best model = 2.22), but temperature was (0.065; 0.0088 - 0.12), with more visits occurring at higher temperatures.

**Table 2.** *Candidate models used to investigate predictors of* B. terrestris audax *strawberry flower visitation in the June-bearer and everbearer crops. The chosen predictors were colony status, strawberry variety and temperature. The null model included only the intercept as a predictor, but included the same random effects structure as all other candidate models. Models are presented from the optimal model with the lowest AICc to the model with the highest AICc at the bottom. The optimal model and those within <2ΔAICc are highlighted in bold. When more than 1 model is highlighted, model-averaging was performed to obtain estimates.*

**Figure 1.** *The flower visitation rate (visits hour^-1^) of* B. terrestris audax *on strawberry flowers in the June-bearing crop. The median (central horizontal line), quartiles (box), non-outlier ranges (vertical lines) and raw data (dots) are presented on the plot.*

**Figure 2.** *The flower visitation rate (visits hour^-1^) of* B. terrestris audax *on strawberry flowers in the everbearing crop. The median (central horizontal line), quartiles (box), non-outlier ranges (vertical lines) and raw data (dots) are presented on the plot.*

**3.2. All pollinator visitation**

In the June-bearers, a total of 574 strawberry flower visits were observed over the course of 27 hours of transects walks. Coleoptera of the family Nitidulidae were the most abundant flower visitors (n=325), followed by Muscoidea Anthomyiidae (n=103), and Apidae (n=94). Syrphidae (n=26) and ‘other Diptera’ (n=22) were scarcer. ‘Other Muscidae’ (n=2), Dermaptera (n=1), and Aphididae (n=1) were the least abundant flower visitors. Within the family Apidae, most of the visits were made by *B. terrestris* (n=86), with only four made by *Apis mellifera*. The mean (± SE) wild pollinator visitation rate was (no. of wild pollinator visits = all flower visits – *B. terrestris audax* visits) 18.08 (± 2.66) visits/hour.

In the everbearers, 5176 flower visits were observed over 48 hours of transect walks. Again, Nitidulidae were by far the most abundant flower visitors (n=4378), followed by Diptera (other) (n=231), Muscoidea Anthomyiidae (n=222), Apidae (n=135), Syrphidae (n=76), Empididae (n=63), Coleoptera (other) (n=45), Stratiomyidae (n=10), Formicidae (n=6), Lepidoptera (n=4), Muscidae (n=3), Andrenidae (n=1), Oedemeridae (n=1), and unknown (n=1). Once again, within the family Apidae, *B. terrestris* was the dominant flower visitor (n=95), with only eighteen *A. mellifera* visits recorded. The mean (± SE) wild pollinator visitation rate was 105.92 (± 26.02) visits/hour.

*B. terrestris audax* visitation was not a strongly supported predictor of wild pollinator visitation in both the June- and everbearing crops (June-bearer: 0.059; -0.092 – 0.21, everbearer: ΔAICc to best model = 2.41).

**3.3. Strawberry quality**

**3.3.1. Berry weight and diameter**

In the June-bearer crop, model-averaged parameter estimates indicated that colony status was not a strong predictor of fruit weight or diameter (weight: ΔAICc to best model = 2.08, diameter: -0.11; -0.40 – 0.18). The growth position of the fruit received strong support as a predictor of both fruit weight and diameter. This variable appeared in all the Δ2AICc set of models for both weight and diameter. As expected, berries from secondary and tertiary growth positions were lighter and smaller than those from primary growth positions (**secondary**: weight: -0.21; -0.25 – -0.17, diameter: -0.98; -1.19 – -0.76, **tertiary**: weight: -0.39; -0.46 – -0.33, diameter: -1.97; -2.29 – -1.64). The variety of the fruit was also a strong predictor of weight and diameter (weight: -0.13; -0.20 – -0.062, diameter: -0.59; -0.90 – -0.28), with berries of the Malling centenary variety being smaller and lighter than Flair.

In the everbearer crop, colony status was not a good predictor of weight (weight: -0.03; -0.16 – 0.10), or diameter (diameter: ΔAICc to best model = 2.05). Growth position featured in all the Δ2AICc models indicating that it was a good predictor of both fruit weight and size. Berries from secondary and tertiary growth positions were lighter and smaller (**secondary:** weight: -0.84; -0.98 – -0.69, diameter: -3.80; -4.71 – -2.88, **tertiary:** weight: -2.04; -2.33 – -1.74, diameter: -12.10; -13.95 – -10.24). Wild pollinator abundance and Nitidulidae beetle abundance had a negative effect on fruit weight and diameter (**wild pollinator:** weight: -0.023; -0.039 – -0.0078, diameter: -0.13; -0.23 – -0.031, **Nitidulidae beetle:** weight: -0.0013; -0.0025 – -0.00011, diameter: -0.0072; -0.014 – -0.00035).

**3.3.2. Achene ratio**

Colony status was not a strong predictor of the proportion of fertilised achenes on a fruit in the June-bearer crop (0.068; -0.015 – 0.15). The variety of the fruit was a good predictor (0.11; 0.0053 – 0.22). In the everbearer crop, colony status was also not a well-supported predictor of the proportion of fertilised achenes (ΔAICc to best model = 2.05).

**3.3.3. Strawberry Class**

In the June-bearer crop, the interaction between colony status and variety was a well-supported predictor of fruit quality classification, featuring in both of the Δ2AICc models (Table 3). This indicated that the treatment had differential effects on fruit quality between the two strawberry varieties. Malling Centenary benefitted from the presence of commercial bumblebees, producing 25% more high commercial grade berries when colonies were open (Figure 3). However, the variety Flair did not receive a benefit from the presence of commercial bumblebees (Figure 4).

In the everbearer crop, colony status was not a well-supported predictor of strawberry quality (Figure 5), featuring in none of the model averaged Δ2AICc models (Table 3; ΔAICc to best model = 2.02). The only well-supported predictor was the growth position of the fruit, with fruit from secondary growth positions being of lower quality (-1.58; -2.01 – -1.14).

**Table 3.** *Model-averaged models* (*the optimal model and those models within <2ΔAICc*) *used to investigate the best predictors of fruit commercial grade in the June-bearer and everbearer crops. Each row of the table represents a unique model. + symbols indicate the inclusion of that covariate in the model. Models including all the predictor variables were tested. The null model included only the intercept as a predictor, but included the same random effects structure as all other candidate models.*

**Figure 3.** *Proportion of fruits within each commercial grade from each treatment in the June-bearing crop variety ‘Malling Centenary’.*

**Figure 4.** *Proportion of fruits within each commercial grade from each treatment in the June-bearing crop variety ‘Flair’.*

**Figure 5.** *Proportion of fruits within each commercial grade from each treatment in the everbearing crop.*

**3.4. Colony weight and activity**

Colony weight and activity did not differ between the colonies used in the June- and everbearer experiments (weight: *p* = 0.81, activity: *p* = 0.53).

**4. Discussion**

To our knowledge, this is the first study investigating the effect of commercial bumblebees on strawberry crop quality in a commercial farm setting in the UK. With commercial bumblebees being used on a variety of crop types, studies like this are essential to verify the assumed benefits that bees provide to the crop. The results indicate that the addition of commercial *B. terrestris audax* colonies to a strawberry crop can have differential effects depending upon the crop variety and its growing season. The June-bearing variety Malling Centenary produced 25% more high commercial grade (extra and class 1) strawberries when commercial bumblebees were able to forage on the crop compared to when they were not able to forage. Malling Centenary is a very popular variety among UK growers (Harnden pers.comm., 2019), thus, commercial bumblebees are likely to be significantly contributing to the economic value of the UK June-bearer strawberry crop. However, the June-bearing variety ‘Flair’ and the later flowering everbearing variety received no benefit from the presence of commercial bumblebees, which raises questions about commercial bumblebee use in all strawberry varieties during all growing seasons.

There are likely to be several factors that are influencing the results we observed. One of the main drivers impacting the effectiveness of commercial bumblebees in the June- and everbearing strawberry crops, is likely to be the differing visitation rates of *B. terrestris audax* on strawberry flowers in these crops. In the June-bearer crop, there were considerably more *B. terrestris audax* visits when colonies were open compared to when they were closed, which suggests that these visits were being made by the commercial bees. This is important because previous studies have suggested that commercial bees may predominantly forage on alternative flowers to the target crop (Foulis and Goulson, 2014; Lye et al., 2011; Murray et al., 2013; Whittington et al., 2004). Our results suggest that this may not be the case when little other forage is available early in the season. During the time periods when the colonies were closed, very few *B. terrestris audax* visits were observed in the June-bearers. Wild bumblebees are still establishing nests and in the early stages of colony development at this time of year (March - April), indeed 35.3% of the total flower visits made by wild bumblebees in the June-bearer crop were made by newly emerged queens. Consequently, it was not surprising to observe low numbers of wild *B. terrestris audax* in the June-bearer polytunnels.

In the everbearing crop, there was very little difference in the *B. terrestris audax* visitation rate between when colonies were open and closed. Even when colonies were open, the *B. terrestris audax* visitation rate to the crop was much lower (1.88 ± 0.34 visits/hour) than the equivalent periods in the June-bearer crop (5.26 ± 0.85 visits/hour). This suggests that the commercial bumblebees in the everbearer crop were infrequently foraging on strawberry flowers, which could have contributed to the lack of effect that commercial bumblebees had in the everbearer crop. At the time of flowering of the everbearer crop (May - June), there were likely to be abundant foraging resources other than the strawberry flowers (Balfour et al., 2018). Thus, commercial bumblebees may have left the strawberry crop in favour of alternate foraging resources, as has been observed in other studies (Foulis and Goulson, 2014; Lye et al., 2011; Murray et al., 2013; Whittington et al., 2004). Furthermore, the wild *B. terrestris audax* population is likely to have been higher during the time of the everbearer experiment, as this time period coincides with when wild colonies are reaching their peak. This is likely to have contributed to the lack of difference seen between visitation rates when colonies were open and closed in the everbearer crop. At the time of flowering of the June-bearer crop (March-April), far fewer alternative foraging resources would have been available in the surrounding environment (Balfour et al., 2018), thus, the commercial bumblebees would have little other choice than to forage on the strawberry crop.

In addition to *B. terrestris* visitation rate, differences between the strawberry varieties themselves are likely to have contributed to the observed results. Strawberry varieties are known to have different dependencies on insect flower visitation to set high quality fruit (Klatt et al., 2014; Zebrowska, 1998). Variety was not a strong predictor of *B. terrestris audax* visitation indicating that the June-bearer varieties were visited at similar rates. Despite this, Flair did not respond to the presence of commercial bumblebees with any improvements in pollination or quality measures, whilst Malling Centenary responded to the commercial bumblebees with a 25% increase in high commercial grade fruit. It is possible that the June-bearing variety ‘Flair’ does not benefit greatly from insect visitation, and thus, the commercial bumblebees were providing no additional benefit. Alternatively, it may require a much higher number of visits than it was receiving. These explanations are also applicable to the everbearer variety. During the time when the everbearers were flowering (May-June) wild pollinators were much more abundant on strawberry flowers. We believe that this makes it more likely that the crop was already sufficiently pollinated by the wild pollinator community, so the commercial bees were not providing any additional benefit to the crop.

In addition to pollination dependencies, different strawberry varieties can vary in the sugar concentration of their nectar, and in the floral volatiles they produce, both of which could alter their attractiveness to pollinators (Abrol, 1992; Klatt et al., 2013). Thus, it is possible that the everbearing variety used in this experiment was not highly attractive to bumblebees both wild and commercial, causing it to receive a lower *B. terrestris audax* visitation rate than the June-bearer varieties. We believe the two June-bearer varieties to have been similarly attractive, as variety was not a strong predictor of *B. terrestris audax* visitation rate.

The architecture of the polytunnels in which the strawberries were grown may have influenced the results. Following standard practice, during the June-bearer experiment, the polythene on the sides of the tunnels was rolled down and only the ends of the tunnels were open, but in the everbearer polytunnels both the sides and ends were open. This may have made it more difficult for commercial bees to leave, and for wild pollinators to enter and disperse amongst, the June-bearer crop (Ellis et al., 2017), which could have contributed to the increased visitation rate, and in the case of Malling Centenary, effectiveness of commercial bees in the June-bearing crop. This suggests that it may be the interaction between crop management practices and commercial bumblebees, rather than the provision of commercial bumblebees alone, that drive potential pollination benefits.

Another factor that could have affected the results is that commercial bumblebees may have foraged on strawberry fields outside of the one in which the colony was placed i.e. bumblebees could have left a field in which all the colonies were open, and foraged in a field in which the commercial colonies were closed, as some of the fields were within *B. terrestris audax* foraging range of each other. However, we do not believe this happened at a large scale. In the June-bearer experiment we saw significant differences in visitation rate between the open and closed colony treatments, which we would not have observed if commercial bumblebees were foraging in adjacent fields in large numbers. Furthermore, it is not clear if *B. terrestris audax* would travel a greater distance (e.g. into a different field) to collect the same quality resource (strawberry). Finally, *B. terrestris audax* mainly fly at <3m above the ground (Osborne et al., 1999) and the windbreaking hedges separating all fields were >5m, which may have deterred bumblebees from crossing to adjacent fields.

It is noteworthy that despite the interaction between colony status and variety being a strong predictor of strawberry shape classification in the June-bearers, it was not a strong predictor of the other fruit variables measured (weight, diameter and achene ratio). Previous studies have found such measures to be highly correlated (Hodgkiss et al., 2018; Klatt et al., 2014). Our results do not necessarily disagree with these previous studies, as the improvement in quality in the Malling Centenary crop was accompanied by small increases in strawberry weight, size and fertilised achene ratio. It appears that these changes on their own were not large enough for colony status to have become a significant predictor of these strawberry measures. In addition, other studies have found correlations between these measures to be weak or in some cases non-existent, meaning that our results are not unprecedented (Herbertsson et al., 2017; Tuohimetsä et al., 2014).

Our results bear some similarities with those of Trillo et al. (2018). Here, they found that the use of commercial bumblebees increased the strawberry flower visitation rate in winter in southern Spain. This is comparable to the increased flower visitation rate we observed on deployment of commercial bumblebees in the June-bearer crop during March and April. Both studies also found that despite increased flower visitation, there was no evidence of competition between commercial bumblebees and other pollinators, suggesting that such large-scale crop flower blooms may provide sufficient foraging resources for pollinators. Furthermore, in southern Spain, they found that commercial bumblebees did not improve the weight or quality of fruit, as seen in the Flair and everbearer crop in our study. The results from Trillo et al. (2018), in combination with those presented here, raise questions about commercial bumblebee use in strawberry crops. Their use incurs financial cost for farmers and can have negative environmental consequences. Thus, commercial bumblebees should only be used when they are providing a significant benefit to the crop, which appears not to be the case in some European growing systems. Clearly further research is required to understand whether commercial bumblebee supplementation is necessary for various strawberry varieties in different growing seasons.

However, the positive effect of commercial bumblebees on fruit quality in the Malling Centenary crop clearly demonstrates the utility of commercial bumblebees when used appropriately. Malling Centenary is a popular UK variety, thus, a 25% increase in high grade fruit represents a large increase in value of the UK strawberry crop. Given the significant benefits of using commercial bumblebees for this variety, greater emphasis should be placed on reducing the negative impacts associated with the trade in commercial colonies, so that they can be used with minimal risk to environmental health.

In contrast with Trillo et al. (2018) and several other studies on different crop types, our results provide no evidence that wild pollinators are improving the crop (Blaauw and Isaacs, 2014; Garibaldi et al., 2013; Greenleaf and Kremen, 2006; Holzschuh et al., 2012). Wild pollinator flower visitation was not a well-supported predictor of fruit quality in the June-bearer crop. This could be explained by the relatively low numbers of wild pollinators present during this time of the year not being sufficient to impact any fruit pollination measures. In the everbearer crop, there was even a negative effect of wild pollinator flower visitation on fruit weight and diameter, despite there being much greater wild pollinator flower visitation rates than in the June-bearer crop. A factor contributing to this could be that the crop was not pollen limited for the duration of the everbearer experiment, even at times when wild pollinator abundances were at their lowest. If this was the case, then additional visits would not be providing any benefit to the crop, and could potentially even be decreasing the quality of the crop by overpollination (Velthuis and van Doorn 2006; Mommaerts, Put, and Smagghe 2011).

Of all the strawberry flower visitors, Coleoptera of the Nitidulidae family were by far the most abundant in the June-bearing and everbearing crops. The majority, if not all of these, were pollen and nectar feeding beetles of the genus *Meligethes.* Given their dominance in the system, we treated them as a separate covariable, to investigate whether their abundance was a predictor of strawberry pollination. Recent evidence suggests that pollen beetles (*Meligethes* spp.) can be a pest on strawberry crops by causing a reduction in fruit weight (Castle et al., 2019). Our results from the everbearer crop, where pollen beetles were particularly abundant, support this, with the beetle flower visitation having a negative effect on fruit weight and size.

It should be noted that this experiment did not cover the entire flowering period of the everbearer crop, which can continue into the late summer months (September/October). It is possible that commercial bumblebees may become of more use later in the season, as wild pollinator abundance and alternate foraging resources decrease (Balfour et al., 2018; Hallmann et al., 2017). Furthermore, other locations may have more depauperate pollinator communities than our study farm, and studies have shown that pollinator communities can drastically change from year to year (Kremen et al., 2002). In such cases, commercial bumblebees may be of use as an insurance policy for crop pollination. Further studies are required to investigate whether the effects observed here vary over space and time, but this study provides a detailed baseline from which to build further studies.

**5. Conclusion**

As commercial bumblebees are increasingly marketed for a broad range of crop types, studies like this one are essential to prove the bees are having a beneficial effect, and to inform growers of how to use commercial bumblebees in an environmentally responsible and cost-effective manner. A 25% increase in high commercial grade fruit represents a significant increase in the value of the Malling Centenary strawberries produced. Thus, the results support the widespread use of commercial bumblebees in this strawberry variety. However, commercial bees placed in the June-bearing variety Flair and an everbearing crop appear to be providing no benefit to fruit quality, indicating that it may not be worth using them in some strawberry varieties and during some parts of the growing season. Based on these results, growers should consider both the strawberry variety and the season in which it is grown, before deploying commercial bumblebees on a farm. However, to allow growers to make fully informed decisions, further research is needed on the pollination requirements of strawberry varieties, and the contribution to crop pollination provided by wild pollinators.

**Acknowledgements**

We would like to thank Jon Rix at Kelsey Farm for allowing the field work to be carried out there, and Richard Harnden, Dylan Hodgkiss, Ash Samuelson, Adrian Harris, Fabio Manfredini, Elli Leadbeater, Arran Folly, Judit Bagi, Emily Bailes and Harry Siviter for advice and assistance. We are grateful to the editor and anonymous reviewers for comments which led to an improved manuscript. This study was funded by a BBSRC CASE studentship BB/L016001/1 to M.J.F.B in collaboration with NIAB EMR and Berry Gardens.

**Author Contributions**

C.D.M, M.T.F and M.J.F.B conceived the initial idea and designed the experiment. C.D.M performed the experiment and statistical analyses. C.D.M wrote the manuscript draft and C.D.M, M.T.F and M.J.F.B provided the final edit.

**Data Availability Statement**

The dataset generated and analysed during the current study is available from the corresponding author upon request. If the manuscript is accepted for publication in *Agriculture, Ecosystems and Environment*, the dataset generated and analysed during this study will be released on a public repository.

**References**

Abrol, D.P., 1992. Energetics of nectar production in some strawberry cultivars as a predictor of floral choice by honeybees. J. Biosci. 17, 41–44. https://doi.org/10.1007/BF02716772

Ahrenfeldt, E.J., Klatt, B.K., Arildsen, J., Trandem, N., Andersson, G.K.S., Tscharntke, T., Smith, H.G., Sigsgaard, L., 2015. Pollinator communities in strawberry crops – variation at multiple spatial scales. Bull. Entomol. Res. 105, 497–506. https://doi.org/10.1017/S000748531500036X

Aizen, M.A., Garibaldi, L.A., Cunningham, S.A., Klein, A.M., 2009. How much does agriculture depend on pollinators? Lessons from long-term trends in crop production. Ann. Bot. 103, 1579–88. https://doi.org/10.1093/aob/mcp076

Balfour, N.J., Ollerton, J., Castellanos, M.C., Ratnieks, F.L.W., 2018. British phenological records indicate high diversity and extinction rates among late-summer-flying pollinators. Biol. Conserv. 222, 278–283. https://doi.org/10.1016/J.BIOCON.2018.04.028

Bartoń, K., 2017. MuMIn: Multimodel inference. Package version 1.40.0.

Bates, D., Maechler, M., Bolker, B., Walker, S., 2017. lme4: linear mixed-effects models using Eigen and S4. Package version 1.1-12.

Berger, L.A., Vaissiére, B.E., Moffett, J.O., Merritt, S.J., 1988. *Bombus* spp. (Hymenoptera: Apidae) as pollinators of male-sterile upland cotton on the Texas high plains. Environ. Entomol. 17, 789–794. https://doi.org/10.1093/ee/17.5.789

Blaauw, B.R., Isaacs, R., 2014. Flower plantings increase wild bee abundance and the pollination services provided to a pollination-dependent crop. J. Appl. Ecol. 51, 890–898. https://doi.org/10.1111/1365-2664.12257

Bolker, B.M., Brooks, M.E., Clark, C.J., Geange, S.W., Poulsen, J.R., Stevens, M.H.H., White, J.-S.S., 2009. Generalized linear mixed models: a practical guide for ecology and evolution. Trends Ecol. Evol. 24, 127–135. https://doi.org/10.1016/J.TREE.2008.10.008

Calderone, N.W., 2012. Insect pollinated crops, insect pollinators and US agriculture: trend analysis of aggregate data for the period 1992–2009. PLoS One 7, e37235. https://doi.org/10.1371/journal.pone.0037235

Castle, D., Grass, I., Westphal, C., 2019. Fruit quantity and quality of strawberries benefit from enhanced pollinator abundance at hedgerows in agricultural landscapes. Agric. Ecosyst. Environ. 275, 14–22. https://doi.org/10.1016/j.agee.2019.01.003

Christensen, R.H.B., 2017. Ordinal: regression models for ordinal data. Version 2015.6-28.

Colla, S.R., Otterstatter, M.C., Gegear, R.J., Thomson, J.D., 2006. Plight of the bumble bee: pathogen spillover from commercial to wild populations. Biol. Conserv. 129, 461–467. https://doi.org/10.1016/j.biocon.2005.11.013

Connelly, H., Poveda, K., Loeb, G., 2015. Landscape simplification decreases wild bee pollination services to strawberry. Agric. Ecosyst. Environ. 211, 51–56. https://doi.org/10.1016/J.AGEE.2015.05.004

DEFRA, 2015. Horticulture Statistics 2015. Dep. Environ. Food Rural Aff.

Desjardins, È.-C., De Oliveira, D., 2006. Commercial bumble bee *Bombus impatiens* (Hymenoptera: Apidae) as a pollinator in lowbush blueberry (Ericale: Ericaceae) fields. J. Econ. Entomol. 99, 443–449. https://doi.org/10.1603/0022-0493-99.2.443

Dimou, M., Taraza, S., Thrasyvoulou, A., Vasilakakis, M., 2008. Effect of bumble bee pollination on greenhouse strawberry production. J. Apic. Res. 47, 99–101. https://doi.org/10.1080/00218839.2008.11101433

Dogterom, M.H., Matteoni, J.A., Plowright, R.C., 1998. Pollination of greenhouse tomatoes by the North American *Bombus vosnesenskii* (Hymenoptera: Apidae). J. Econ. Entomol. 91, 71–75. https://doi.org/10.1093/jee/91.1.71

Eilers, E.J., Kremen, C., Smith Greenleaf, S., Garber, A.K., Klein, A.-M., 2011. Contribution of pollinator-mediated crops to nutrients in the human food supply. PLoS One 6, e21363. https://doi.org/10.1371/journal.pone.0021363

Ellis, C.R., Feltham, H., Park, K., Hanley, N., Goulson, D., 2017. Seasonal complementary in pollinators of soft-fruit crops. Basic Appl. Ecol. 19, 45–55. https://doi.org/10.1016/J.BAAE.2016.11.007

European Commission, 2011. Commission Regulation (EU) No 543/2011.

FAOSTATS: Food and Agricultural Organaziation of the United Nations. 2016 Statistical division. See http://faostat.fao.org/default.aspx., n.d.

Feltham, H., Park, K., Minderman, J., Goulson, D., 2015. Experimental evidence that wildflower strips increase pollinator visits to crops. Ecol. Evol. 5, 3523–30. https://doi.org/10.1002/ece3.1444

Foulis, E.S.J., Goulson, D., 2014. Commercial bumble bees on soft fruit farms collect pollen mainly from wildflowers rather than the target crops. J. Apic. Res. 53, 404–407. https://doi.org/10.3896/IBRA.1.53.3.08

Garibaldi, L.A., Steffan-Dewenter, I., Winfree, R., Aizen, M.A., Bommarco, R., Cunningham, S.A., Kremen, C., Carvalheiro, L.G., Harder, L.D., Afik, O., Bartomeus, I., Benjamin, F., Boreux, V., Cariveau, D., Chacoff, N.P., Dudenhöffer, J.H., Freitas, B.M., Ghazoul, J., Greenleaf, S., Hipólito, J., Holzschuh, A., Howlett, B., Isaacs, R., Javorek, S.K., Kennedy, C.M., Krewenka, K.M., Krishnan, S., Mandelik, Y., Mayfield, M.M., Motzke, I., Munyuli, T., Nault, B.A., Otieno, M., Petersen, J., Pisanty, G., Potts, S.G., Rader, R., Ricketts, T.H., Rundlöf, M., Seymour, C.L., Schüepp, C., Szentgyörgyi, H., Taki, H., Tscharntke, T., Vergara, C.H., Viana, B.F., Wanger, T.C., Westphal, C., Williams, N., Klein, A.-M., 2013. Wild pollinators enhance fruit set of crops regardless of honey bee abundance. Science. 339, 1608–11. https://doi.org/10.1126/science.1230200

Goka, K., Okabe, K., Yoneda, M., 2006. Worldwide migration of parasitic mites as a result of bumblebee commercialization. Popul. Ecol. 48, 285–291. https://doi.org/10.1007/s10144-006-0010-8

Goodell, K., Thomson, J.D., 2007. Influence of bee species (Hymenoptera: Apiformes) with contrasting behaviors on pollen movement in a mustard, *Brassica rapa* (Brassicaceae) and the muskmelon *Cucumis melo* (Cucurbitaceae). Entomol. Gen. 29, 237–252. https://doi.org/10.1127/entom.gen/29/2007/237

Graystock, P., Yates, K., Evison, S.E.F., Darvill, B., Goulson, D., Hughes, W.O.H., 2013. The Trojan hives: pollinator pathogens, imported and distributed in bumblebee colonies. J. Appl. Ecol. 50, 1207–1215. https://doi.org/10.1111/1365-2664.12134

Greenleaf, S.S., Kremen, C., 2006. Wild bee species increase tomato production and respond differently to surrounding land use in Northern California. Biol. Conserv. 133, 81–87. https://doi.org/10.1016/J.BIOCON.2006.05.025

Hallmann, C.A., Sorg, M., Jongejans, E., Siepel, H., Hofland, N., Schwan, H., Stenmans, W., Müller, A., Sumser, H., Hörren, T., Goulson, D., de Kroon, H., 2017. More than 75 percent decline over 27 years in total flying insect biomass in protected areas. PLoS One 12, e0185809. https://doi.org/10.1371/journal.pone.0185809

Herbertsson, L., Gåvertsson, I., Klatt, B.K., Smith, H.G., 2017. Assessing the risk of stigma clogging in strawberry flowers due to pollinator sharing with oilseed rape. J. Pollinat. Ecol. 21, 71–77.

Hicks, B.J., Sircom, J., 2016. Pollination of commercial cranberry (*Vaccinium macrocarpon* Ait.) by native and introduced managed bees in Newfoundland. J. Acadian Entomol. Soc. 12, 22–30.

Hodgkiss, D., Brown, M.J.F., Fountain, M.T., 2018. Syrphine hoverflies are effective pollinators of commercial strawberry. J. Pollinat. Ecol. 22, 55–66.

Holzschuh, A., Dudenhöffer, J.-H., Tscharntke, T., 2012. Landscapes with wild bee habitats enhance pollination, fruit set and yield of sweet cherry. Biol. Conserv. 153, 101–107. https://doi.org/10.1016/J.BIOCON.2012.04.032

Ings, T.C., Ward, N.L., Chittka, L., 2006. Can commercially imported bumble bees out-compete their native conspecifics? J. Appl. Ecol. 43, 940–948. https://doi.org/10.1111/j.1365-2664.2006.01199.x

Inoue, M.N., Yokoyama, J., Washitani, I., 2008. Displacement of Japanese native bumblebees by the recently introduced *Bombus terrestris* (L.) (Hymenoptera: Apidae). J. Insect Conserv. 12, 135–146. https://doi.org/10.1007/s10841-007-9071-z

Javorek, S.K., Mackenzie, K.E., Kloet, S.P. Vander, 2002. Comparative pollination effectiveness among bees (Hymenoptera: Apoidea) on lowbush blueberry (Ericaceae: *Vaccinium angustifolium*). Ann. Entomol. Soc. Am. 95, 345–351. https://doi.org/10.1603/0013-8746(2002)095[0345:CPEABH]2.0.CO;2

Johnson, J.B., Omland, K.S., 2004. Model selection in ecology and evolution. Trends Ecol. Evol. 19, 101–108. https://doi.org/10.1016/J.TREE.2003.10.013

Klatt, B.K., Burmeister, C., Westphal, C., Tscharntke, T., von Fragstein, M., 2013. Flower volatiles, crop varieties and bee responses. PLoS One 8, e72724. https://doi.org/10.1371/journal.pone.0072724

Klatt, B.K., Holzschuh, A., Westphal, C., Clough, Y., Smit, I., Pawelzik, E., Tscharntke, T., 2014. Bee pollination improves crop quality, shelf life and commercial value. Proc. R. Soc. B-Biological Sci. 281. https://doi.org/10.1098/rspb.2013.2440

Klein, A.-M., Vaissière, B.E., Cane, J.H., Steffan-Dewenter, I., Cunningham, S.A., Kremen, C., Tscharntke, T., 2007. Importance of pollinators in changing landscapes for world crops. Proc. R. Soc. B-Biological Sci. 274, 303–313. https://doi.org/10.1098/rspb.2006.3721

Kremen, C., Williams, N.M., Thorp, R.W., 2002. Crop pollination from native bees at risk from agricultural intensification. Proc. Natl. Acad. Sci. U. S. A. 99, 16812–16816. https://doi.org/10.1073/pnas.262413599

Lye, G.C., Jennings, S.N., Osborne, J.L., Goulson, D., 2011. Impacts of the use of nonnative commercial bumble bees for pollinator supplementation in raspberry. J. Econ. Entomol. 104, 107–114. https://doi.org/10.1603/ec10092

MacInnis, G., Forrest, J.R.K., 2019. Pollination by wild bees yields larger strawberries than pollination by honey bees. J. Appl. Ecol. https://doi.org/10.1111/1365-2664.13344

Mommaerts, V., Put, K., Smagghe, G., 2011. *Bombus terrestris* as pollinator-and-vector to suppress *Botrytis cinerea* in greenhouse strawberry. Pest Manag. Sci. 67, 1069–1075. https://doi.org/10.1002/ps.2147

Murray, T.E., Coffey, M.F., Kehoe, E., Horgan, F.G., 2013. Pathogen prevalence in commercially reared bumble bees and evidence of spillover in conspecific populations. Biol. Conserv. 159, 269–276. https://doi.org/10.1016/J.BIOCON.2012.10.021

Osborne, J.L., Clark, S.J., Morris, R.J., Williams, I.H., Riley, J.R., Smith, A.D., Reynolds, D.R., Edwards, A.S., 1999. A landscape-scale study of bumble bee foraging range and constancy, using harmonic radar. J. Appl. Ecol. 36, 519–533. https://doi.org/10.1046/j.1365-2664.1999.00428.x

Petersen, J.D., Reiners, S., Nault, B.A., 2013. Pollination services provided by bees in pumpkin fields supplemented with either *Apis mellifera* or *Bombus impatiens* or not supplemented. PLoS One 8, e69819. https://doi.org/10.1371/journal.pone.0069819

R Core Team, 2018. R: a language and environment for statistical computing. R Foundation for Statistical Computing, Vienna, Austria.

Rasmont, P., Coppee, A., Michez, D., De Meulemeester, T., 2008. An overview of the *Bombus terrestris* (L. 1758) subspecies (Hymenoptera: Apidae). Ann. la Société Entomol. Fr. 44, 243–250. https://doi.org/10.1080/00379271.2008.10697559

Sapir, G., Baras, Z., Azmon, G., Goldway, M., Shafir, S., Allouche, A., Stern, E., Stern, R.A., 2017. Synergistic effects between bumblebees and honey bees in apple orchards increase cross pollination, seed number and fruit size. Sci. Hortic. (Amsterdam). 219, 107–117. https://doi.org/10.1016/J.SCIENTA.2017.03.010

Schmid-Hempel, R., Eckhardt, M., Goulson, D., Heinzmann, D., Lange, C., Plischuk, S., Escudero, L.R., Salathe, R., Scriven, J.J., Schmid-Hempel, P., 2014. The invasion of southern South America by imported bumblebees and associated parasites. J. Anim. Ecol. 83, 823–837. https://doi.org/10.1111/1365-2656.12185

Shipp, J.L., Whitfield, G.H., Papadopoulos, A.P., 1994. Effectiveness of the bumble bee, *Bombus impatiens* Cr. (Hymenoptera: Apidae), as a pollinator of greenhouse sweet pepper. Sci. Hortic. (Amsterdam). 57, 29–39. https://doi.org/10.1016/0304-4238(94)90032-9

Skaug, H., Fournier, D., Bolker, B., Magnusson, A., Nielson, A., 2016. Generalized linear mixed models using ’AD model. R package version 0.8.3.3.

Stanghellini, M., Ambrose, J., Schultheis, J., 1998. Seed production in watermelon: a comparison between two commercially available pollinators. HortScience 33, 28–30.

Stanghellini, M., Ambrose, J., Schultheis, J., 1997. The effects of honey bee and bumble bee pollination on fruit set and abortion of cucumber and watermelon. Am. Bee J. 137, 386–391.

Stubbs, C.S., Drummond, F.A., 2001. *Bombus impatiens* (Hymenoptera: Apidae): an alternative to *Apis mellifera* (Hymenoptera: Apidae) for lowbush blueberry pollination. J. Econ. Entomol. 94, 609–616. https://doi.org/10.1603/0022-0493-94.3.609

Taylor, L.R., 1963. Analysis of the effect of temperature on insects in flight. J. Anim. Ecol. 32, 99–117. https://doi.org/10.2307/2520

Thomson, J.D., Goodell, K., 2002. Pollen removal and deposition by honeybee and bumblebee visitors to apple and almond flowers. J. Appl. Ecol. 38, 1032–1044. https://doi.org/10.1046/j.1365-2664.2001.00657.x

Trillo, A., Herrera, J.M., Vilà, M., 2018. Managed bumble bees increase flower visitation but not fruit weight in polytunnel strawberry crops. Basic Appl. Ecol. 30, 32–40. https://doi.org/10.1016/J.BAAE.2018.05.008

Tuohimetsä, S., Hietaranta, T., Uosukainen, M., Kukkonen, S., Karhu, S., 2014. Fruit development in artificially self- and cross-pollinated strawberries ( *Fragaria* × *ananassa* ) and raspberries ( *Rubus idaeus* ). Acta Agric. Scand. Sect. B — Soil Plant Sci. 64, 408–415. https://doi.org/10.1080/09064710.2014.919348

van Ravestijn, W., Nederpel, L., 1988. Trostrillers in België aan de kant: hommels doen het werk. Groenten en Fruit 6, 38–41.

Velthuis, H.H.W., van Doorn, A., 2006. A century of advances in bumblebee domestication and the economic and environmental aspects of its commercialization for pollination. Apidologie 37, 421–451. https://doi.org/10.1051/apido:2006019

Wang, X.-H., Ding, S.-Y., 2012. Pollinator-dependent production of food nutrients by fruits and vegetables in China. African J. Agric. Res. 7, 6136–6142. https://doi.org/10.5897/AJAR12.1764

Webb, R.A., Purves, J. V., White, B.A., 1974. The components of fruit size in strawberry. Sci. Hortic. (Amsterdam). 2, 165–174. https://doi.org/10.1016/0304-4238(74)90006-5

Whittington, R., Winston, M.L., Tucker, C., Parachnowitsch, A.L., 2004. Plant-species identity of pollen collected by bumblebees placed in greenhouses for tomato pollination. Can. J. Plant Sci. 84, 599–602. https://doi.org/10.4141/P02-192

Wietzke, A., Westphal, C., Gras, P., Kraft, M., Pfohl, K., Karlovsky, P., Pawelzik, E., Tscharntke, T., Smit, I., 2018. Insect pollination as a key factor for strawberry physiology and marketable fruit quality. Agric. Ecosyst. Environ. 258, 197–204. https://doi.org/10.1016/J.AGEE.2018.01.036

Zebrowska, J., 1998. Influence of pollination modes on yield components in strawberry (*Fragaria x ananassa* Duch.). Plant Breed. 117, 255–260. https://doi.org/10.1111/j.1439-0523.1998.tb01935.x

Zhang, H., Huang, J., Williams, P.H., Vaissière, B.E., Zhou, Z., Gai, Q., Dong, J., An, J., 2015. Managed bumblebees outperform honeybees in increasing peach fruit set in China: different limiting processes with different pollinators. PLoS One 10, e0121143. https://doi.org/10.1371/journal.pone.0121143

**Supplementary material**

**Supplementary figure S1.** *Land use classification of the area surrounding the farm. The circular classified area has a diameter of 2.75km with the centre point being in the middle of the farm. This diameter was chosen as it included the land use up to 1km away from all the commercial bumblebee colonies. Definitions of each land use class and the area they occupy can be found in supplementary tables S2 and S1 respectively.*

**Supplementary figure S2.** *Layout of colonies and fields in the June-bearer experiment. The fields sampled are shaded in blue. The green blocks indicate sampling areas; the polytunnels from which strawberries were sampled. Each green block consists of three tunnels: one central tunnel which contained the commercial bumblebee colony, and the two tunnels either side of this central tunnel. The northernmost sampling areas in the southernmost field and in the central field, only consist only of 2 polytunnels. This was done to take into account the differing sizes of the polytunnels (see section ‘2. Methodology’ for details).*

**Supplementary figure S3.** *Layout of colonies and fields in the everbearer experiment. The fields sampled are shaded in red. The green blocks indicate sampling areas; the polytunnels from which strawberries were sampled. Each green block consists of three tunnels: one central tunnel which contained the commercial bumblebee colony, and the two tunnels either side of this central tunnel.*

**Supplementary figure S4.** *An example of a recently opened strawberry flower that was tagged in the experiment.*

**Supplementary figure S5.** *Examples of strawberries from each commercial grade classification. Strawberry colour was not taken into account in the quality assessment, as berries often had to be harvested before fully ripened.*

**Supplementary table S1.** *The area covered (m^2^) by each land use in a 2.75km diameter circle centred on the farm. This diameter was chosen as it includes the land cover within a 1km radius of every commercial bumblebee colony that was placed on the farm. Definitions of each land use class can be found in supplementary table S2.*

**Supplementary table S2.** *Definitions of each land use classification.*

**Supplementary table S3.** *The strawberry variety grown, the size, and the commercial bumblebee colony density within each sampling area from which fruit were picked. The position of each sampling area can be seen in supplementary figures 2 and 3. Fields prefixed with ‘JB’ were sampled in the June-bearer experiment, and those prefixed with ‘EV’ are from the everbearer experiment.*

**Supplementary table S4.** *Model-averaged models* (*the optimal model and those models within <2ΔAICc*) *used to investigate the best predictors of strawberry weight, strawberry diameter, proportion of fertilised achenes, and strawberry class in the June-bearer strawberry crop. + symbols indicate the inclusion of that covariate in the model. Models including all combinations of the predictor variables were tested. The null model included only the intercept as a predictor, but included the same random effects structure as all other candidate models.*

**Supplementary table S5.** *Model-averaged models* (*the optimal model and those models within <2ΔAICc*) *used to investigate the best predictors of strawberry weight, strawberry diameter, proportion of fertilised achenes, and strawberry class in the everbearer strawberry crop. + symbols indicate the inclusion of that covariate in the model. Models including all combinations of the predictor variables were tested. The null model included only the intercept as a predictor, but included the same random effects structure as all other candidate models.*
