# Supplementary material for: Varietal and seasonal differences in the effects of commercial bumblebees on fruit quality in strawberry crops
Source: Agric Ecosyst Environ. 2019 Sep 1;281:124–33. doi: 10.1016/j.agee.2019.04.007 (PMC6686987; doi:10.1016/j.agee.2019.04.007)
Supplement: Supplementary file 2 [file mmc2.pdf]

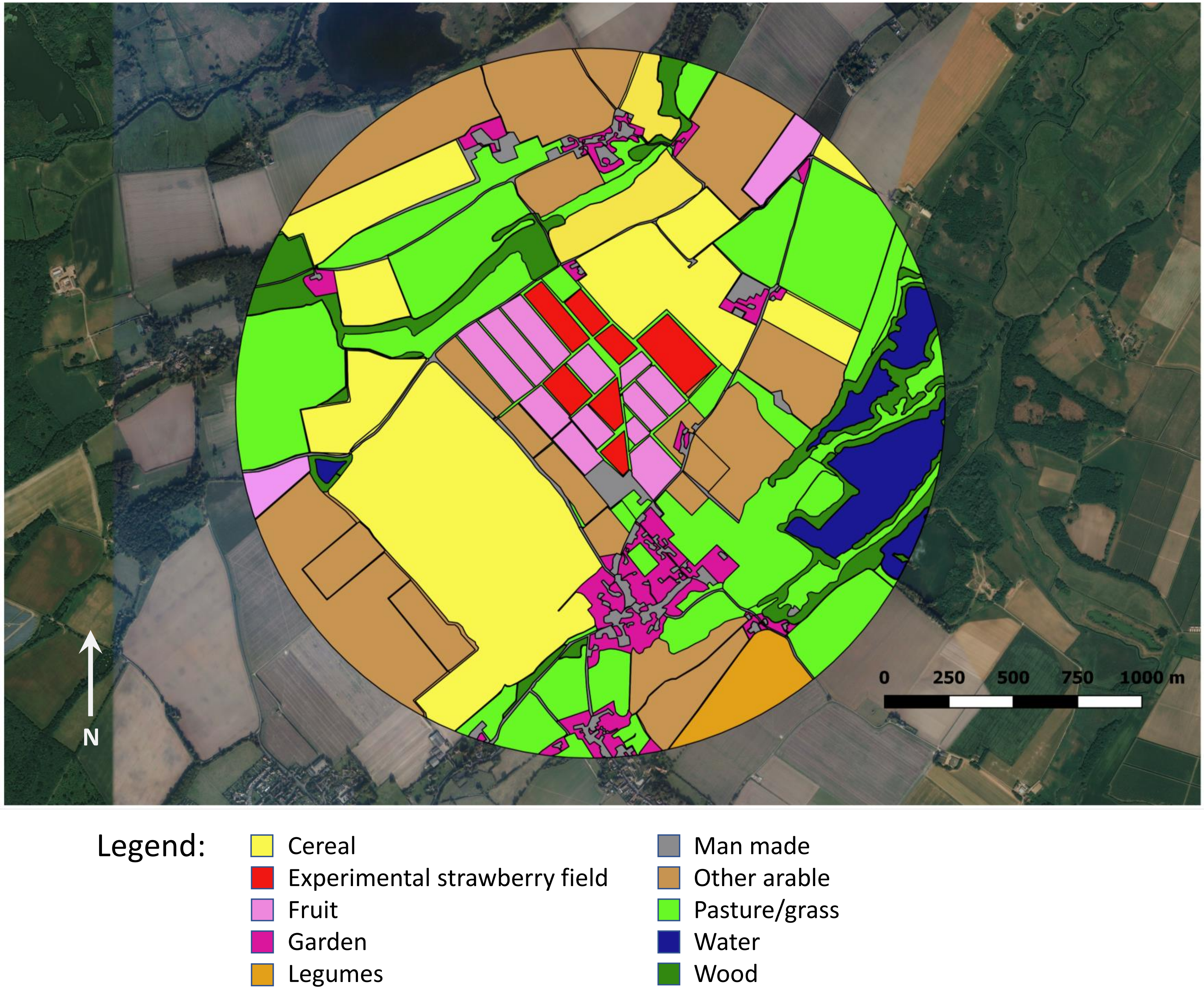
0
250
500
750
1000 m
N
Legend:
Cereal
Experimental strawberry field
Fruit
Garden
Legumes
Man made
Other arable
Pasture/grass
Water
Wood
