# Supplementary figures and images for: Varietal and seasonal differences in the effects of commercial bumblebees on fruit quality in strawberry crops
Source: Agric Ecosyst Environ. 2019 Sep 1;281:124–33. doi: 10.1016/j.agee.2019.04.007 (PMC6686987; doi:10.1016/j.agee.2019.04.007)

## Slide 1
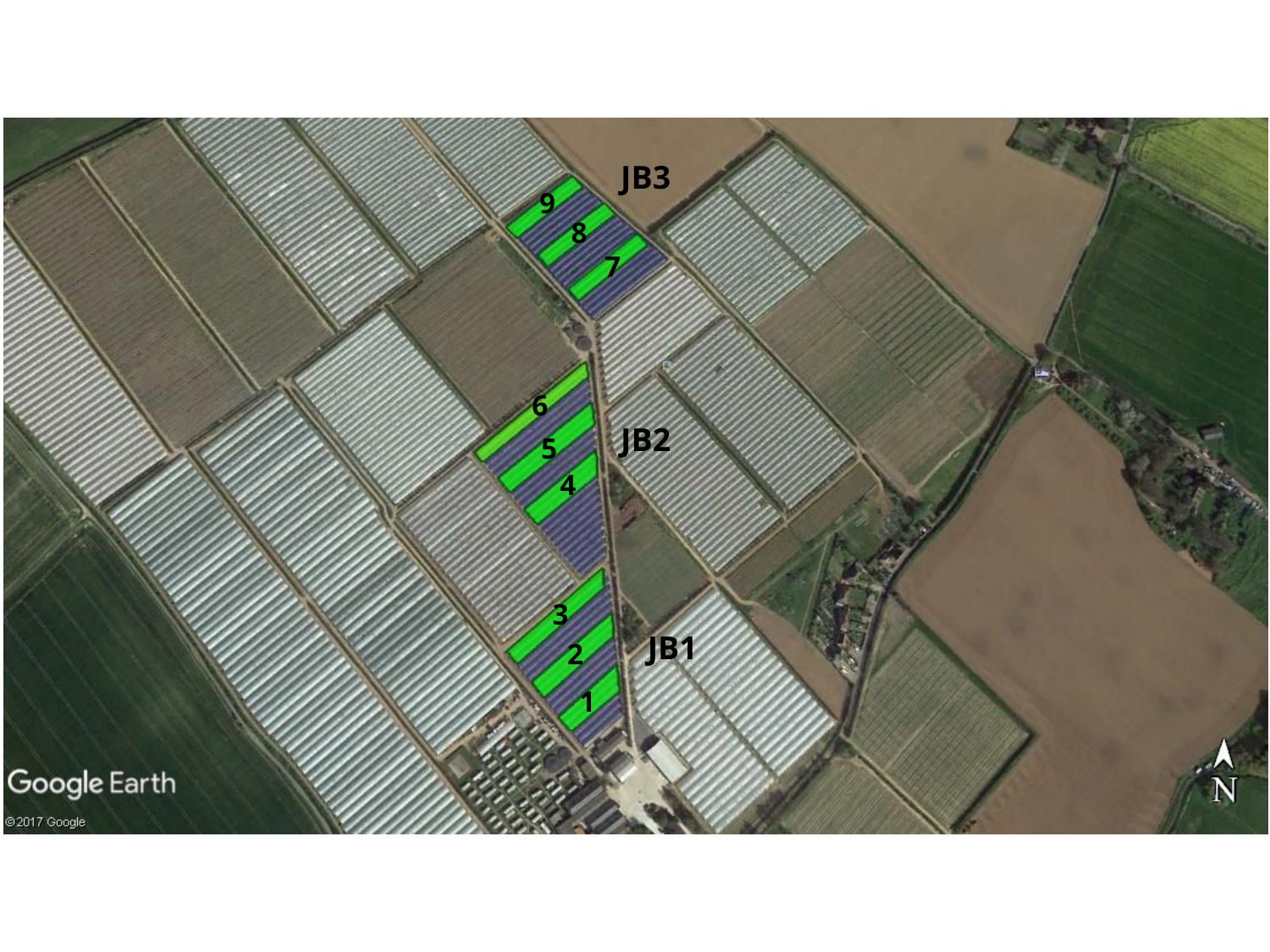

JB3
9
8
7
6
JB2
5
4
3
JB1
2
1

Supplement: Supplementary file 3 [file mmc3.pptx]

## Slide 1
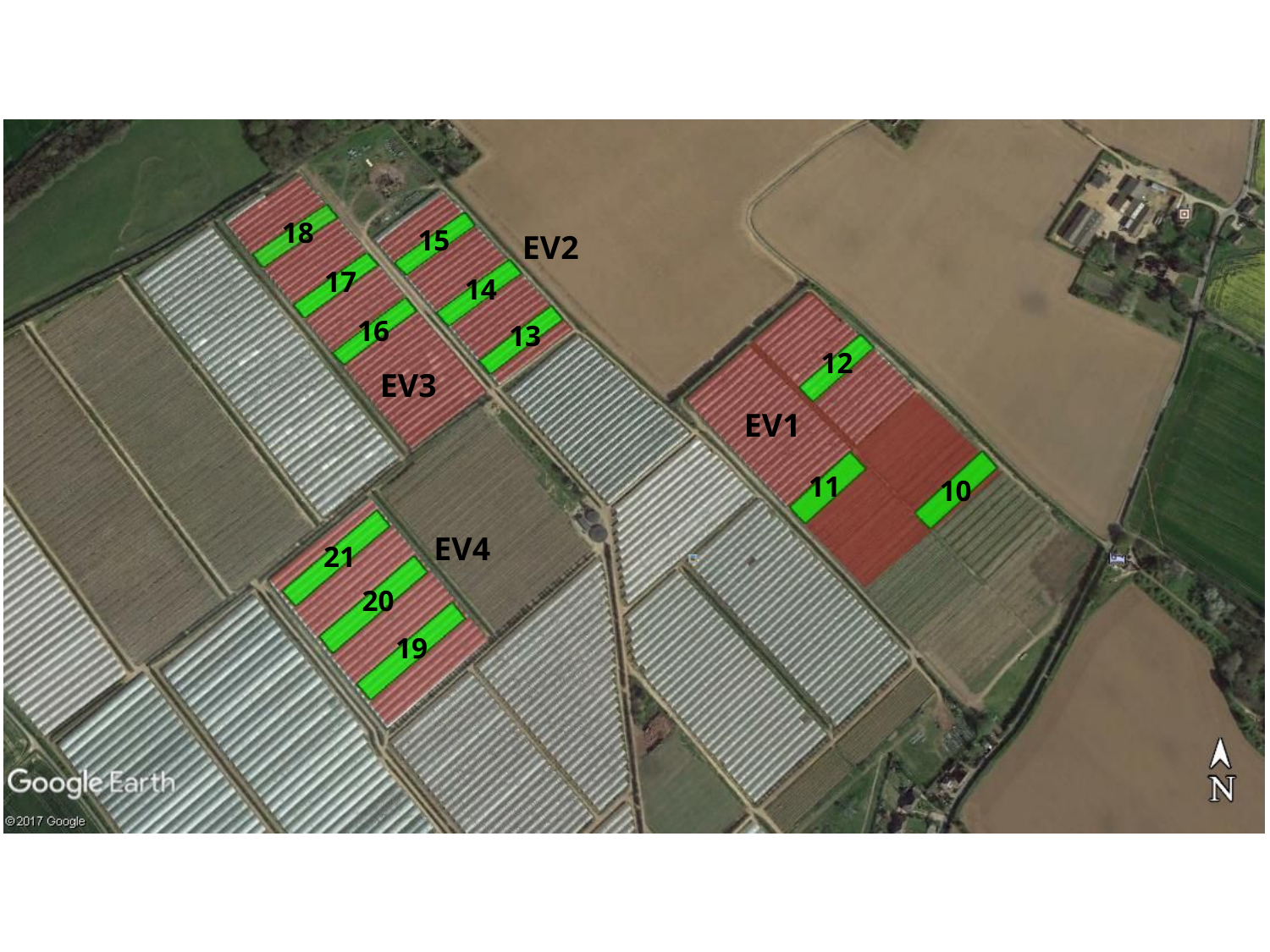

18
15
EV2
17
14
16
13
12
EV3
EV1
11
10
EV4
21
20
19

Supplement: Supplementary file 4 [file mmc4.pptx]

## Slide 1
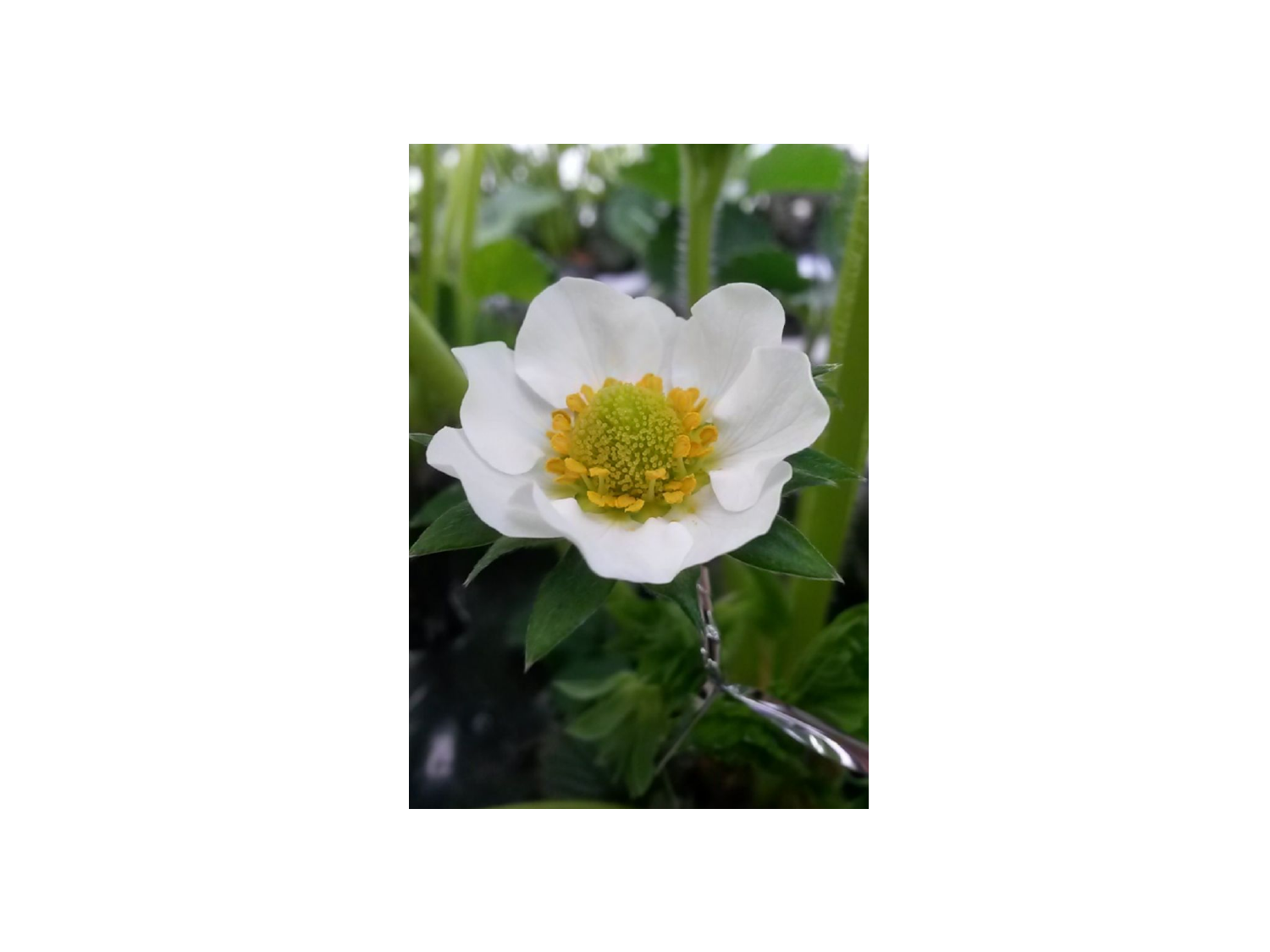

Supplement: Supplementary file 5 [file mmc5.pptx]

## Slide 1
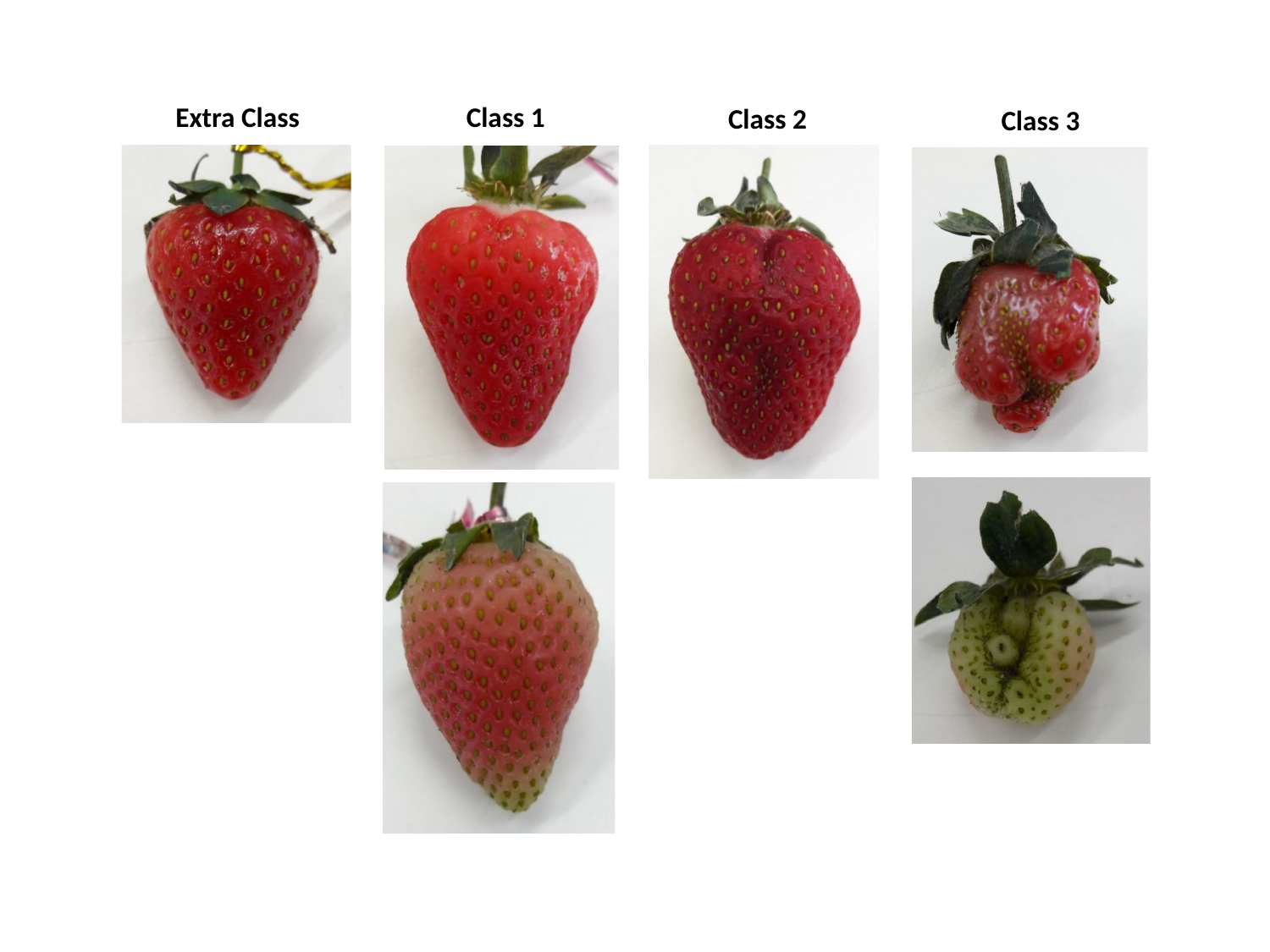

Extra Class
Class 1
Class 2
Class 3

Supplement: Supplementary file 6 [file mmc6.pptx]
