# Supplementary material for: Varietal and seasonal differences in the effects of commercial bumblebees on fruit quality in strawberry crops
Source: Agric Ecosyst Environ. 2019 Sep 1;281:124–33. doi: 10.1016/j.agee.2019.04.007 (PMC6686987; doi:10.1016/j.agee.2019.04.007)
Supplement: Supplementary file 7 [file mmc7.docx]

**Supplementary table S1.** *The area covered (m^2^) by each land use in a 2.75km diameter circle centred on the farm. This diameter was chosen as it includes the land cover within a 1km radius of every commercial bumblebee colony that was placed on the farm. Definitions of each land use class can be found in supplementary table S2.*

| **Land use** | **Area covered (m^2^)** |
| --- | --- |
| cereal | 1473075 |
| experimental strawberry fields | 140113 |
| fruit | 313824 |
| garden | 216126 |
| legumes | 104329 |
| man made | 232891 |
| other arable | 1333681 |
| pasture/grass | 1544499 |
| water | 230598 |
| wood | 342082 |
