# Supplementary material for: Varietal and seasonal differences in the effects of commercial bumblebees on fruit quality in strawberry crops
Source: Agric Ecosyst Environ. 2019 Sep 1;281:124–33. doi: 10.1016/j.agee.2019.04.007 (PMC6686987; doi:10.1016/j.agee.2019.04.007)
Supplement: Supplementary file 8 [file mmc8.docx]

**Supplementary table S2.** *Definitions of each land use classification.*

| **Land use** | **Description** |
| --- | --- |
| Cereal | Cereal crops (wheat, maize) |
| Experimental strawberry fields | Strawberry fields into which commercial colonies were placed |
| Fruit | Fruit crops (apple, blackcurrant, strawberry, raspberry) |
| Garden | Residential gardens |
| Legumes | Legume crops (field bean) |
| Man made | Roads and buildings |
| Other arable | Arable crops that were not identified |
| Pasture/grass | Grazing pasture and grassland areas that were not residential gardens |
| Water | Areas of water |
| Wood | Woodland (predominantly mixed deciduous) |
