# Supplementary material for: Varietal and seasonal differences in the effects of commercial bumblebees on fruit quality in strawberry crops
Source: Agric Ecosyst Environ. 2019 Sep 1;281:124–33. doi: 10.1016/j.agee.2019.04.007 (PMC6686987; doi:10.1016/j.agee.2019.04.007)
Supplement: Supplementary file 9 [file mmc9.docx]

| **Sampling area** | **Field** | **Strawberry variety** | **Sampling area size (hectares)** | **Colony density within sampling area (colonies/ha)** |
| --- | --- | --- | --- | --- |
| 1 | JB1 | Malling Centenary | 0.160 | 6.250 |
| 2 | JB1 | Malling Centenary | 0.185 | 5.405 |
| 3 | JB1 | Malling Centenary | 0.168 | 5.952 |
| 4 | JB2 | Flair | 0.167 | 5.988 |
| 5 | JB2 | Flair | 0.210 | 4.762 |
| 6 | JB2 | Flair | 0.188 | 5.319 |
| 7 | JB3 | Malling Centenary | 0.181 | 5.525 |
| 8 | JB3 | Malling Centenary | 0.180 | 5.556 |
| 9 | JB3 | Malling Centenary | 0.178 | 5.618 |
| 10 | EV1 | Proprietary variety 1 | 0.165 | 6.061 |
| 11 | EV1 | Proprietary variety 1 | 0.160 | 6.250 |
| 12 | EV1 | Proprietary variety 1 | 0.160 | 6.250 |
| 13 | EV2 | Proprietary variety 1 | 0.165 | 6.061 |
| 14 | EV2 | Proprietary variety 1 | 0.164 | 6.105 |
| 15 | EV2 | Proprietary variety 1 | 0.164 | 6.105 |
| 16 | EV3 | Proprietary variety 1 | 0.163 | 6.154 |
| 17 | EV3 | Proprietary variety 1 | 0.163 | 6.154 |
| 18 | EV3 | Proprietary variety 1 | 0.163 | 6.154 |
| 19 | EV4 | Proprietary variety 1 | 0.200 | 5.000 |
| 20 | EV4 | Proprietary variety 1 | 0.200 | 5.000 |
| 21 | EV4 | Proprietary variety 1 | 0.200 | 5.000 |

**Supplementary table S3.** *The strawberry variety grown, the size, and the commercial bumblebee colony density within each sampling area from which fruit were picked. The position of each sampling area can be seen in supplementary figures 2 and 3. Fields prefixed with ‘JB’ were sampled in the June-bearer experiment, and those prefixed with ‘EV’ are from the everbearer experiment.*
