# Supplementary material for: Varietal and seasonal differences in the effects of commercial bumblebees on fruit quality in strawberry crops
Source: Agric Ecosyst Environ. 2019 Sep 1;281:124–33. doi: 10.1016/j.agee.2019.04.007 (PMC6686987; doi:10.1016/j.agee.2019.04.007)
Supplement: Supplementary file 10 [file mmc10.docx]

| **colony status** | **growth position** | | **wild pollinator abundance** | **pollen beetle abundance** | **variety** | | **colony status:variety** | | **AICc** | **ΔAICc** |
| --- | --- | --- | --- | --- | --- | --- | --- | --- | --- | --- |
| **strawberry weight** | | |  |  |  | |  | |  |  |
|  | + | |  |  | + | |  | | 2488.60 | 0.00 |
|  | + | | + |  | + | |  | | 2489.20 | 0.60 |
|  | + | |  | + | + | |  | | 2489.40 | 0.80 |
|  | + | | + | + | + | |  | | 2490.32 | 1.72 |
| **strawberry diameter** | |  | |  |  |  | |  |  |  |
|  | + | |  |  | + | |  | | 2335.70 | 0.00 |
|  | + | |  | + | + | |  | | 2336.58 | 0.88 |
|  | + | | + |  | + | |  | | 2336.80 | 1.10 |
| + | + | |  |  | + | |  | | 2337.62 | 1.92 |
| + | + | |  | + | + | |  | | 2337.62 | 1.92 |
| **achene ratio** | | |  |  |  | |  | |  |  |
|  | + | |  |  | + | |  | | 1594.70 | 0.00 |
|  |  | |  |  | + | |  | | 1595.49 | 0.79 |
| + | + | |  |  | + | |  | | 1596.03 | 1.33 |
|  | + | |  |  |  | |  | | 1596.04 | 1.34 |
|  | + | |  | + | + | |  | | 1596.59 | 1.89 |
| **strawberry class** | | |  |  |  | |  | |  |  |
| + |  | |  | + | + | | + | | 501.70 | 0.00 |
| + |  | |  |  | + | | + | | 502.20 | 0.50 |

**Supplementary table S4.** *Model-averaged models* (*the optimal model and those models within <2ΔAICc*) *used to investigate the best predictors of strawberry weight, strawberry diameter, proportion of fertilised achenes, and strawberry class in the June-bearer strawberry crop. + symbols indicate the inclusion of that covariate in the model. Models including all combinations of the predictor variables were tested. The null model included only the intercept as a predictor, but included the same random effects structure as all other candidate models.*
