# Supplementary material for: Varietal and seasonal differences in the effects of commercial bumblebees on fruit quality in strawberry crops
Source: Agric Ecosyst Environ. 2019 Sep 1;281:124–33. doi: 10.1016/j.agee.2019.04.007 (PMC6686987; doi:10.1016/j.agee.2019.04.007)
Supplement: Supplementary file 11 [file mmc11.docx]

**Supplementary table S5.** *Model-averaged models* (*the optimal model and those models within <2ΔAICc*) *used to investigate the best predictors of strawberry weight, strawberry diameter, proportion of fertilised achenes, and strawberry class in the everbearer strawberry crop. + symbols indicate the inclusion of that covariate in the model. Models including all combinations of the predictor variables were tested. The null model included only the intercept as a predictor, but included the same random effects structure as all other candidate models.*

| **colony status** | **growth position** | **wild pollinator abundance** | **pollen beetle abundance** | **field identity** | **AICc** | **ΔAICc** |
| --- | --- | --- | --- | --- | --- | --- |
| **strawberry weight** | |  |  |  |  |  |
|  | + | + | + | + | 5826.90 | 0.00 |
|  | + | + | + |  | 5827.01 | 0.11 |
|  | + | + |  |  | 5828.52 | 1.62 |
| + | + | + | + | + | 5828.55 | 1.65 |
| + | + | + | + |  | 5828.57 | 1.67 |
|  | + | + |  | + | 5828.66 | 1.76 |
| **strawberry diameter** | | |  |  |  |  |
|  | + | + | + | + | 5291.90 | 0.00 |
|  | + | + | + |  | 5292.48 | 0.58 |
|  | + | + |  | + | 5293.26 | 1.36 |
|  | + | + |  |  | 5293.39 | 1.49 |
| **achene ratio** | |  |  |  |  |  |
|  | + |  | + | + | 3138.30 | 0.00 |
|  | + | + | + | + | 3140.03 | 1.73 |
| **strawberry class** | |  |  |  |  |  |
|  | + |  |  |  | 817.80 | 0.00 |
|  | + | + |  |  | 818.36 | 0.56 |
|  | + |  | + |  | 819.56 | 1.76 |
